# 502 Estimation of Return to Work After Burn Injury

**DOI:** 10.1093/jbcr/irac012.133

**Published:** 2022-03-23

**Authors:** Aidan Ifland, Naiwei Hsu, Matthew J Reiss

**Affiliations:** Torrance Memorial Medical Center, Torrance, California; Torrance Memorial Medical Center, Torrance, California; Torrance Memorial Medical Center, Torrance, California

## Abstract

**Introduction:**

A major milestone of recovery from a burn injury is returning to work (RTW). A systematic review by Mason et al. suggested that perhaps 28% of burn survivors do not return to work, while other studies have identified complex factors that affect RTW, including patient age, pre-existing mental health issues, injury locations such as hands, and type of work they do. Due to the large number of complex interactions involved, predicting a timeline for RTW is challenging.

We anticipate that underestimations of RTW may result in frustration, and perhaps contribute to negative psychological outcomes. Therefore, we wished to exam the accuracy of our surgeons estimates of RTW.

**Methods:**

A retrospective chart review of inpatients treated at our center with burn injury, with Worker's Compensation as insurance, with follow up at our outpatient clinic, and admitted during the calendar years 2018 to 2019, produced 23 records with sufficient documentation. Hospital admission was taken as the starting point, and number of days until the (first) estimated RTW date was compared to the number of days until the actual date of RTW if documented, or else the final documented surgeons estimate.

**Results:**

Limited demographic information and results are found in table 1. in 6 cases (23%), patients had not returned to work by one-year post hospitalization. Reasons for non-return to work in all cases included chronic pain requiring a pain management specialist, or psychologic sequelae requiring psychological treatment, or both.

We found that our surgeons estimated an average RTW date 65 days post admission date, whereas the average actual RTW was 177 days after admission. Expressed as a ratio, the actual time was 2.9x the estimated time, and expressed as a difference, the surgeons underestimated by 112 days.

**Conclusions:**

The actual time needed for RTW was much longer that the surgeons initial assessment. These findings suggest that despite a wealth of experience in burn care, surgeons are prone to underestimate recovery time and especially time to RTW.

**Table:**
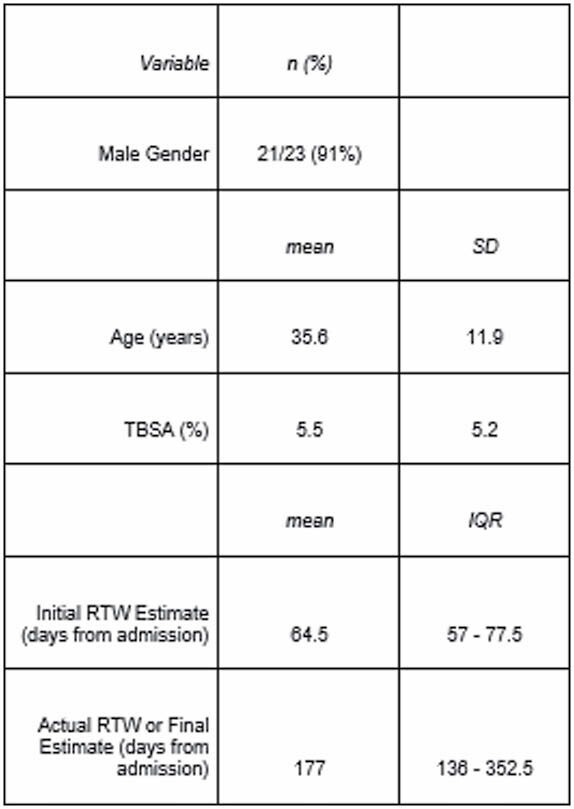
Table 1 - Demographics and Results. SD = standard deviation, IQR = interquatile range, TBSA = Total Body Surface Area burned.

